# Acute Stress Decreases but Chronic Stress Increases Myocardial Sensitivity to Ischemic Injury in Rodents

**DOI:** 10.3389/fpsyt.2016.00071

**Published:** 2016-04-25

**Authors:** Eric D. Eisenmann, Boyd R. Rorabaugh, Phillip R. Zoladz

**Affiliations:** ^1^Department of Psychology, Sociology and Criminal Justice, Ohio Northern University, Ada, OH, USA; ^2^Department of Pharmaceutical and Biomedical Sciences, Ohio Northern University, Ada, OH, USA

**Keywords:** stress, cardiovascular, ischemia, anxiety, PTSD, rodent

## Abstract

Cardiovascular disease (CVD) is the largest cause of mortality worldwide, and stress is a significant contributor to the development of CVD. The relationship between acute and chronic stress and CVD is well evidenced. Acute stress can lead to arrhythmias and ischemic injury. However, recent evidence in rodent models suggests that acute stress can decrease sensitivity to myocardial ischemia–reperfusion injury (IRI). Conversely, chronic stress is arrhythmogenic and increases sensitivity to myocardial IRI. Few studies have examined the impact of validated animal models of stress-related psychological disorders on the ischemic heart. This review examines the work that has been completed using rat models to study the effects of stress on myocardial sensitivity to ischemic injury. Utilization of animal models of stress-related psychological disorders is critical in the prevention and treatment of cardiovascular disorders in patients experiencing stress-related psychiatric conditions.

## Introduction

The goal of this review is to analyze recent literature utilizing rodent models to examine the impact of psychological stress on sensitivity to myocardial ischemia–reperfusion injury (IRI) in the context of the well-established relationship between stress, myocardial ischemic injury, and cardiovascular disease (CVD). Stress is a general adaptive response provoked by stimuli that disrupt homeostasis (1, 2). The stress response activates systems responsible for mobilizing the energy and resources necessary to overcome this homeostatic disturbance. The main systems activated include the hypothalamic–pituitary–adrenal (HPA) axis and the sympathetic adrenomedullary (SAM) system (3, 4). Stress results in the release of corticotropin-releasing hormone (CRH) from the paraventricular nucleus, which then causes the release of adrenocorticotropic hormone (ACTH) from the anterior pituitary. ACTH acts on the adrenal cortex to synthesize and secrete the glucocorticoid (GC) hormone cortisol (in humans) or corticosterone (in rodents) (3, 5). The hypothalamus also activates the adrenal medulla *via* the sympathetic nervous system (SNS), which results in the release of the catecholamines epinephrine and norepinephrine. ACTH, CRH, and GCs provide the negative feedback necessary to dampen the stress response and return the body to homeostasis (4, 6). Cessation of the stress response is important to prevent damage associated with a prolonged stress response (3, 4, 7). Acute stress generally results in an adaptive response to homeostatic changes; the stress response becomes harmful if it persists chronically (8–11). Thus, stress research can be roughly divided into research examining the effects of acute or chronic stress (3, 4, 7, 9–11).

Physical or psychological stressors can result in the stress response. Physical stressors disrupt the internal or external environment of an organism and include stimuli such as anoxia, heat, cold, or physical strain (exercise or injury). Psychological stressors are stimuli that affect emotion and result in fear, anxiety, or frustration (8–11). As previously discussed, anything disrupting homeostasis can be a stressor; however, this review focuses on stressors with a psychological component.

Chronic stress can have damaging effects on the whole organism (4). Stress precipitates psychiatric disease, such as depression and post-traumatic stress disorder (PTSD), and worsens physical health outcomes, such as CVD (12, 13). Furthermore, patients with psychiatric disorders have a higher incidence of CVD and cardiovascular risk factors, such as atherosclerosis, hypertension, and myocardial infarction (MI) (14–16). Patients with psychiatric disorders experience worse outcomes in response to cardiovascular disorders (e.g., higher mortality). It is suggested that appropriate monitoring for psychiatric disorders could improve outcomes in patients with ischemic heart disease (8, 14, 17–21). Thus, research directed at minimizing the negative impact of stress is important (19, 21–25).

### Stress and Cardiovascular Disease

Cardiovascular disease is the leading cause of mortality worldwide (26, 27), and stress is a well-established contributor to the development of CVD (3, 8, 20). Stress is relevant at all stages of CVD; stress can increase exposure to risk factors for CVD (e.g., smoking), the long-term development of atherosclerosis, and the triggering of cardiac events in people with CVD (28).

The most common form of CVD is ischemic heart disease (also known as coronary artery disease), which includes disease states such as angina, MI, and sudden cardiac death (SCD) (29, 30). MI occurs when blood flow to a region of the heart stops. The heart is an electromechanical pump; SCD most commonly occurs in response to ventricular fibrillation, a disturbance in electrical activity, as a result of acute coronary ischemia (31, 32). MI and SCD can lead to cardiac arrest and death. Stress may acutely trigger MI or SCD or worsen underling CVD leading to one of these events (3). Thus, stress is closely related to ischemic heart disease. Research investigating the relationship between stress and the cardiovascular system is critical to improve patient outcomes in CVD (20, 25, 28).

#### Myocardial Ischemia–Reperfusion Injury

Myocardial IRI refers to the damage created by the stoppage of and the subsequent restoration of blood flow to the heart. Without blood flow, an imbalance between oxygen supply and demand is created which results directly in irreversible damage to cardiac tissue, eventually resulting in apoptosis or necrosis; this oxygen imbalance is referred to as ischemia. The duration of ischemia and amount of tissue exposed to ischemia are well established as the primary determinants of infarct size (IS), or the amount of non-viable tissue following ischemia. The mechanisms by which damage and protection occur in response to myocardial IRI has been described in detail previously (33–39). Thus, myocardial IRI is the primary mechanism by which cardiac tissue is damaged in MI, SCD, cardiac bypass surgery, and organ transplantation (40). Acute and chronic stress has an impact on myocardial IRI (3, 41, 42). Because myocardial IRI plays a major role in the morbidity and mortality associated with ischemic heart disease and MI, direct study of this pathology is desirable (35, 43–46). To better elucidate the mechanisms underlying CVD and ischemic injury, researchers have utilized animal models.

### The Utility of Animal Models in Stress Biology and Cardiovascular Disease

Animal models are used extensively to study the relationship between stress and CVD. Animal models are especially important in studying stress biology, as they allow researchers to standardize the conditions of stress. Furthermore, a high level of experimental control and the potential to study causal neurobiological and behavioral mechanisms (with easier access to tissue samples and physiological manipulation) makes animal models advantageous for studying cardiovascular function and stress (22, 47, 48). By using validated methodology with translational relevance to human patients, researchers can use animal models effectively to examine underlying mechanisms and potential treatment options in CVD and stress (22, 49).

#### The Langendorff Isolated Heart – An Experimental Model of Ischemic Injury

Animal models have been developed to experimentally induce and study acute ischemia both *in vivo* (50, 51) and *ex vivo* (44, 52, 53). The Langendorff isolated heart preparation is one of the most extensively used animal models for the study of heart physiology and ischemia (53). In this model, crystalloid perfusates (or blood) is delivered through a cannula inserted in the ascending aorta. Retrograde flow closes the leaflets in the aortic valve, leading to perfusion of the coronary vasculature (52, 53). This model is commonly used to study myocardial IRI. This is accomplished by occlusion of a coronary artery (typically the left anterior descending artery), leading to regional ischemia, or by turning off flow, leading to global ischemia. This model allows the generation of data including IS, the recovery of contractile function, and electrical activity in response to induced ischemia. In regional ischemia, researchers use the IS relative to the area at risk (AAR), or the area normally perfused by the clamped artery, whereas global ischemia allows measurement of the total amount of non-viable tissue [for a complete methodological review of the Langendorff isolated heart, see Ref. (52)].

Notably, the Langendorff isolated heart system studies ischemic injury in the absence of normal humoral or neuronal stimulation, potentially limiting the translation of experimental findings to the clinical setting (52, 53). Furthermore, this model has additional disadvantages, including a high coronary flow rate, limited supply of high-energy phosphate, a reduced oxygen requirement, and a degree of technical skill required to perform successfully (53–55). These disadvantages have led to the development of alternative methods to study cardiovascular injury; other potentially more clinically relevant methods include altering the Langendorff procedure (54) or using *in vivo* models of cardiovascular injury (56). Despite its disadvantages, the Langendorff isolated heart system has proven invaluable to the study of myocardial IRI (52, 53). This model has been used effectively to identify potential strategies and pharmacological agents to decrease the amount of damage caused to the heart following MI (43, 53).

##### The Langendorff Isolated Heart Preparation in Rats

The Langendorff heart preparation is appropriate in mammalian species. Although this preparation has been used rarely in large animals or man (57–61), the most frequently used isolated heart model is that of the rat. The rat model allows for relatively low costs, easy handling, and uncomplicated equipment (53). Furthermore, the consistency of limited collateral circulation allows the study of regional ischemia in the rat. This provides an advantage over models with significant collateralization such as dog (62), guinea-pig (62, 63), and hamster models (63). Furthermore, the rat’s consistent coronary structure makes it a better model than, for example, rabbits, whose coronary structure varies significantly between animals (64). However, it is important to recognize that the rat suffers distinct disadvantages in cardiovascular study because of its short action potential duration, which lacks a plateau phase. This makes this animal a poor choice for study of arrhythmogenesis and antiarrhythmic drugs (60, 65–68). Similarly, dogs have been shown to have elevated levels of troponin and creatine kinase, markers of cardiac damage, in response to cardiac injury (69). However, rats have only shown elevations in troponin, making them relatively poor candidates to study drug-induced injury using these markers (69, 70). Thus, one must remain mindful of the potential clinical relevance of studies in the context of the species being utilized (52).

Both myocardial ischemic injury and cardiovascular responses to stress have been described in detail in both human patients and animal models; however, only several recent studies have focused directly on the sensitivity to myocardial ischemic injury in response following acute or chronic psychological stress exposure.

## Acute Stress and Cardiovascular Disease

The association between acute stress and cardiac rhythm, acute MI, SCD, and stress cardiomyopathy has been supported by epidemiological studies (71–75). Cardiac rhythm changes in response to acute stress has been evidenced by a marked increase in tachyarrhythmia among patients with implanted cardioverter defibrillators in the New York area of the USA during the attacks on the World Trade Center on September 11, 2001 (71). An association between intense emotional stress or anger and the triggering of acute cardiac events, such as acute MI or SCD, has been demonstrated by multiple studies demonstrating a significant number of patients experiencing an emotional episode roughly 2 h before cardiac arrest (72–75). This increased incidence of MI has been evidenced in individual patients following a significant acute stressor, such as the loss of a loved one. Moreover, acute cardiac event incidence is increased in geographical areas where a major trauma, such as an earthquake, serves as an acute stressor (8, 20, 76). SCD and MIs are rare in patients with no underlying coronary heart disease, whereas stress cardiomyopathy can occur with no underlying disorder (77–79).

### Acute Stress and Myocardial Ischemic Injury

The association between intense emotional stress and ischemic heart disease, specifically the incidence of SCD, has been researched for over 50 years (80, 81). Acute psychological stress in human patients leads to ischemia, stress cardiomyopathy, MI, and SCD (8). Stress cardiomyopathy is induced by intense stress that results in heart weakness without underlying pathology. Thus, stress cardiomyopathy is a recently identified disease state mirroring MI with symptoms, such as chest pain and ECG abnormalities, but without concomitant coronary spasm or ischemia-induced enzymatic release (82, 83). Mental stress elicits regional ischemic damage due to epicardial or microvascular constriction, as evidenced by changes in regional perfusion. Interestingly, this ischemia is not associated with the angina and ECG changes that are associated with exercise-induced stress (84–89). This transient myocardial ischemia and coronary artery constriction have been shown to occur in patients with advanced coronary artery disease in response to mental stress (89–91). Furthermore, mental stress has been shown to lead to ECG alternans, a predictor of ventricular arrhythmias and SCD (92–94).

Acute mental stress has been shown to alter the action potential duration of cardiac tissue in humans. Adrenergic stimulation with isoprenaline and adrenaline increases the steepness of the slope of action potential duration restitution; this suggests that adrenergic stimulation can lead to electrical instability, which could lead to ventricular fibrillation or arrhythmias (95). In an elegant study, Child et al. showed that a mental challenge was able to elicit this effect on action potential duration independent of the respiration or heart rate changes that occur in response to mental stress (96). Ventricular fibrillation has been shown to occur in response to both regional myocardial ischemia and electrical instability. Ventricular fibrillation leads to global cardiac ischemia, which can lead to cardiac death (97, 98). The ability of mental stress to cause cardiac ischemia and electrical instability in the heart is supported by epidemiological studies. The underlying risk factors inherent in clinical study complicate cardiovascular research. As previously discussed, the standardization of stress conditions makes animal models advantageous for investigating the underlying pathology of disease, including CVD.

### Experimental Acute Stress and Cardiovascular Disease

Experimental work using animal models supports the effects of acute psychological stress on the cardiovascular system seen in human patients. Psychological stress has been shown to reduce the ventricular fibrillation threshold in dog (42, 99–103) and porcine models (104). Verrier and colleagues have demonstrated the ability of acute stress to precipitate ventricular arrhythmias in dogs exposed to anger and fear in both healthy hearts and hearts exposed to coronary artery occlusion (99–103, 105–108). Acute stress was able to precipitate ventricular fibrillation and cardiac arrest; albeit, these studies did not utilize dogs exposed to a single acute stressor but rather an acute stress session following aversive conditioning (99–101, 103). These researchers found that behaviorally induced changes in vulnerability to fibrillation are mediated by the direct effects of catecholamines on beta receptors (109, 110). Further supporting the centrally mediated nature of cardiac arrhythmias generated by acute stress, Skinner and Reed were able to prevent an increase in ventricular fibrillation by cryogenic blockage of the forebrain, posterior hypothalamus, or fields of Forel (104). Thus, acute psychological stress has the ability to generate and exacerbate ischemia and ventricular arrhythmia.

Stress-limiting endogenous systems have been identified with the ability to abolish or reduce cardiac arrhythmias in response to sympathetic stimulation, acute stress, or ischemic injury (4, 7). The endogenous hormones utilized by these systems with protective effects on the cardiovascular system include GABA (111, 112), opioids (113), or vagal stimulation with cholinergic agonists (114, 115). Furthermore, it has been suggested that electrical instability does not necessarily disturb cardiac contractility (4, 116). Supporting the role of stress-limiting systems in cardiovascular injury, recent work in rodents demonstrates that acute stress may decrease damage in response to induced regional ischemia, possibly as a compensatory mechanism.

#### Experimental Acute Stress and Myocardial Ischemic Injury

Recent rodent studies looking at the effect of acute psychological stress on the impact of myocardial ischemic injury have found acute stress to be cardioprotective and reduce IS [see Table 1 (45, 117)]. The identified relevant studies utilized cold-restraint stress (117) and forced swim stress (45) before using the Langendorff method to induce regional ischemia. Acute swim stress and acute restraint stress are validated psychological stressors that have been used in combination with other stressors to model PTSD and depression (118–121). These stressors, individually or in combination, have resulted in anxiety-like and fear-related behavior in rodents as assessed by tests such as the elevated plus maze (EPM) and contextual fear conditioning (CF) (119, 122, 123). The decreased sensitivity to myocardial IRI provided by acute psychological stress is supported by similar findings in studies utilizing acute physiologic stressors, such as exercise or hyperthermia (124–128). The existence of endogenous signaling pathways that protect the heart from ischemic injury is well evidenced (46, 129–131).

**Table 1:** **Studies examining myocardial ischemic injury in rodent models of psychological stress**.

Subjects	Stress protocol	Reperfusion injury (RI) protocol	Primary finding	Reference
**Acute psychological stress**				
Adult male Wistar rats	Forced swim for 10 min	30 min ischemia	Decreased infarct size (IS)/area at risk (AAR)%	Moghimian et al. (45)
	RI 10 min after	60 min reperfusion		
Adult male Sprague-Dawley rats	Individual immobilization, placed in a cold room for 3 h at 4 ± 0.3°C	30 min ischemia	Decreased IS/AAR%	Wu et al. (117)
	RI immediately after	120 min reperfusion		
**Chronic psychological stress**				
Adult male Sprague-Dawley rats	1–1.5 h daily restraint stress for 8–14 days	30 min ischemia	Increased IS/AAR%	Scheuer and Mifflin (132)
	RI 24 h later	180 min reperfusion	Increased # of fatal arrhythmias	
Adult male Sprague-Dawley rats	2 h daily restraint stress for 11–12 days	30 min ischemia	Increased IS/AAR%	Scheuer and Mifflin (132)
	RI 24 h later	180 min reperfusion	Increased # of fatal arrhythmias	
Adult male Wistar-Kyoto (WKY) rats	Crowding stress (living space 200 cm^2^/rat) for 8 weeks	30 min ischemia	Decreased LVDP recovery	Ravingerova et al. (133)
	RI unspecified	120 min reperfusion (reperfusion-induced tachyarrhythmias and contractile function measured 40 min after reperfusion initiation)	Increased duration of ventricular tachycardia (VT)	
Adult male spontaneously hypertensive (SHR) rats	Crowding stress (living space 200 cm^2^/rat) for 8 weeks	30 min ischemia	Increased LVDP recovery	Ravingerova et al. (133)
	RI unspecified	120 min reperfusion (reperfusion-induced tachyarrhythmias and contractile function measured 40 min after reperfusion initiation)	Decreased duration of VT	
Adult male Wistar rats	10 s electrical shock, 50 s rest for 1 h daily for 7 days	30 min ischemia	Increased IS/AAR%	Rakhshan et al. (10)
	RI 24 h later	120 min reperfusion		
Adult male Wistar rats	Witnessed rats receive but did not receive 10 s electrical shock, 50 s rest for 1 h daily for 7 days (psychological shock)	30 min ischemia	Increased IS/AAR%	Rakhshan et al. (10)
	RI 24 h later	120 min reperfusion		
5-week-old male Wistar-Kyoto (WKY) rats	Crowding stress (~70 cm^2^ living space per 100g body mass) for 14 days	30 min ischemia	No significant difference between stress and no stress groups	Ledvenyiova-Farkasova et al. (134)
	RI unspecified	120 min reperfusion (reperfusion-induced tachyarrhythmias and contractile function measured 40 min after reperfusion initiation)		
5-week-old female Wistar-Kyoto (WKY) rats	Crowding stress (~70 cm^2^ living space per 100 g body mass) for 14 days	30 min ischemia	Decreased VT duration	Ledvenyiova-Farkasova et al. (134)
	RI unspecified	120 min reperfusion (reperfusion-induced tachyarrhythmias and contractile function measured 40 min after reperfusion initiation)		
5-week-old female spontaneously hypertensive (SHR) rats	Crowding stress (~70 cm^2^ living space per 100 g body mass) for 14 days	30 min ischemia	Increased VT duration	Ledvenyiova-Farkasova et al. (134)
	RI unspecified	120 min reperfusion (reperfusion-induced tachyarrhythmias and contractile function measured 40 min after reperfusion initiation)		
5-week-old male spontaneously hypertensive (SHR) rats	Crowding stress (~70 cm^2^ living space per 100 g body mass) for 14 days	30 min ischemia	Increased VT duration	Ledvenyiova-Farkasova et al. (134)
	RI unspecified	120 min reperfusion (reperfusion-induced tachyarrhythmias and contractile function measured 40 min after reperfusion initiation)		
Adult male Sprague-Dawley rats	31 days chronic social instability (randomized paired housing)	20 min ischemia	Increased IS/AAR%	Rorabaugh et al. (135)
	1 h immobilized predator exposure on days 1 and 11	120 min reperfusion	Decreased RPP	
	See Zoladz et al. (136) for complete PTSD paradigm RI 48 h later		Decreased + dP/dT	
Adult female Sprague-Dawley rats	31 days chronic social instability (randomized paired housing)	20 min ischemia	No significant effect	Rorabaugh et al. (135)
	1 h immobilized predator exposure on days 1 and 11	120 min reperfusion		
	See Zoladz et al. (136) for complete PTSD paradigm			
	RI 48 h after			

Research has previously shown that short-term stress is accompanied by enhanced contractile function and resistance to hypoxia in hearts isolated from stressed animals, while long-term stress resulted in the opposite effect (4, 7). Additionally, acute stressors seem to result in the redistribution of the immune system to the site of inflammation, which could provide an adaptive response to stress (137–139). Interestingly, opioid antagonists were able to eliminate the cardioprotection afforded by cold-restraint stress, supporting this stress-limiting system’s role in decreased sensitivity to ischemic damage (113, 117, 140).

Though acute psychological stress decreases the sensitivity of ischemic damage in response to myocardial IRI, the work does not necessarily contradict the previously discussed, ­well-established effects of acute stress in both animal models and clinical research, including triggering MI or independently leading to ischemic damage (72–75, 100–103). While electrical instability of the heart occurs in response to acute stress, it is possible that protective pathways exist to reduce the sensitivity to ischemic damage (4, 7, 116, 140). Additionally, it is important to recognize that while removing the additional stressors and underlying pathology found in humans adds experimental control, it does diminish the clinical translatability of this work (33, 52, 53). Furthermore, while investigators look at the myocardial ischemic injury of all rodents exposed to acute psychological stress, MI data in humans in response to acute stressors typically only represent patients who experienced an MI or symptoms of an MI (72–75). As a final potential limitation, rodent models look at the same ischemic injury in all subjects, whereas human patients can present with very different ischemic damage due to underlying disease and the possible collateralization of vessels over many years (135).

Contrasting the protective effects of acute stress, chronic stress in rodent models has impacted sensitivity to myocardial ischemic injury in rodent models by decreasing recovery of cardiac contractility and increasing ischemic injury (10, 132, 133, 134). The effect of chronic psychological stress is especially relevant because of the numerous stressors facing human patients, which have effects on cardiovascular outcomes (8, 14, 17–22, 141, 142). Thus, diminishing the negative effects of chronic stress on the heart has the ability to reduce cardiovascular morbidity and mortality. Therefore, the effect of chronic stress on the cardiovascular system has been an emerging area of research with several recent studies looking directly at myocardial ischemic injury.

## Chronic Stress and Cardiovascular Disease

Chronic stress has been implicated to cause or worsen CVD in human patients (20, 141–145). Chronic stress has been linked to increased risk of ischemic heart disease (20, 28). The INTERHEART case–control study showed that significant long-term stress over the course of 12 months more than doubled the risk of acute MI, even after adjusting for conventional risk factors such as diabetes mellitus, hypertension, and smoking (146). Prospective cohort studies have supported the effect of long-term stress on risk of coronary heart disease. Studies have linked coronary heart disease risk with work-related stressors, specifically when an imbalance between effort and reward is experienced (147–151). Furthermore, the effects of long-term stress may persist long after the cessation of the chronic stressors. Survivors of the siege of Leningrad were found to have increased blood pressure and increased mortality from CVD, relative to Russians who were not in the besieged city, over 50 years after the event (152).

### Chronic Stress and Cardiovascular Disease

Psychological conditions related to chronic stress and CVD include depression, anxiety, and PTSD (3). As previously discussed, psychiatric disorders can worsen outcomes in CVD. However, this relationship may be bidirectional. For example, it has been shown that coronary heart disease leads to a higher incidence of depression, and depression leads to worse outcomes in coronary heart disease (14, 15, 17, 49, 153). Furthermore, the association between depression and coronary heart disease occurs independent of comorbid risk factors such as high cholesterol, hypertension, or obesity (13, 49, 154, 155). PTSD also increases a patient’s risk for developing coronary heart disease. This association is independent of comorbid depression, genetic influences, and other confounding factors (156–158). The negative cardiovascular outcomes exhibited in both depression and PTSD have been attributed to underlying dysfunction in the autonomic nervous system and HPA axis (13, 22, 48, 49, 135). However, precisely defining the contribution of long-term stress to CVD is difficult due to potential confounding factors including the aforementioned psychological disorders (28). Thus, animal models provide an acceptable means to study chronic stress in the controlled experimental setting (22).

### Experimental Chronic Stress and Cardiovascular Disease

Animal models support the negative effects of chronic stress on the cardiovascular system evidenced by epidemiological studies. Experimental studies have found exposure to chronic stress results in enhanced development of atherosclerosis and plaque destabilization (3, 159, 160). Chronic stress has also been shown to lower the threshold for ventricular arrhythmias (103, 107–109, 161, 162). In a landmark study, Verrier and Lown conditioned dogs to associate a sling with an aversive shock for 3 days. On days 4 and 5, these researchers found that coronary occlusion in dogs re-exposed to the sling environment (in the absence of shock) led to ventricular fibrillation, whereas dogs in a non-aversive cage environment did not experience ventricular fibrillation. Research has continued to focus on this ability of chronic psychological stress to result in cardiac instability (101, 102, 107).

Researchers have used validated models of psychological disorders to study the relationship between psychological disorders and the cardiovascular system. For example, the relationship between depression and CVD has been studied using chronic stress models [e.g., chronic mild stress (CMS) and social isolation] of depression in rodents. The CMS model of depression involves exposure to mild and unpredictable stressors, including changing cage mates, cage tilt, and periods of water or food deprivation, for a period greater than 2 weeks (49, 153, 163). These models of depression decrease rodent intake of a sweet solution, suggestive of anhedonia. Rodents exposed to these well-established animal models display depressive-like behavior, and have a decreased threshold for arrhythmias and tissue fibrosis (22, 49, 153, 163–167). Although animal models have been used to study stress biology and cardiovascular outcomes, few studies exist using validated models of psychological disorders to study the effect of stress on sensitivity to myocardial ischemic injury.

#### Experimental Chronic Stress and Myocardial Ischemic Injury

In several recent rodent studies, researchers have found greater ISs, decreased cardiac output, and decreased recovery of contractile function in response to chronic psychological stress [see Table 1 (10, 132, 133, 134, 135)]. Chronic physiologic stress has previously shown mixed results; both decreased (168) and increased (169) sensitivity to myocardial ischemic injury have been reported. Evidencing only negative effects of chronic stress on myocardial ischemic injury, the impact of chronic psychological stress represents an emerging area of research to minimize the detrimental effect of chronic stress (135, 170). The disruptive effect of chronic psychological stress exposure on myocardial ischemic injury has been demonstrated using several different chronic stressors, including chronic restraint stress (132), daily foot shocks or witnessing rats receiving those foot shocks (10), or crowding stress (133, 134).

These stressors are frequently utilized in modeling psychological disorders that result from stress. Restraint stress has been used as a psychological stressor in rats and has been utilized in combination with other stressors to model PTSD and depression (119, 122, 123, 136). Inescapable footshock is used to model depressive symptoms in rodents. Rats exposed to inescapable footshock have demonstrated anxiety-like behavior on an EPM, impaired growth rates, decreased rearing in an open field, and decreased locomotion (50, 171–173). Crowding stress is a well-known and ethologically valid model of psychological stress in rats which causes social competition for resources, such as space, food, and water. Crowding stress results in behavioral and physiologic data reflecting psychological stress (174–178). These chronic psychological stressors resulted in disruption to the cardiovascular system following induced myocardial ischemic injury, either by causing increased IS and decreased contractile function recovery (10, 132) or only decreased contractile function recovery (133, 134). These studies suggest that chronic stress not only increases the likelihood of a MI or SCD but also exacerbates the damage in response to ischemic injury.

A potential limitation of these studies is that researchers did not take behavioral measures of stress prior to myocardial ischemic injury. Although the methods of stress used to stress these animals are validated as methods of inducing psychological stress, individual susceptibility may play a role in the response of the animal to a psychological stressor (10, 132, 133, 134). Stress exposure may affect animals differently, and thus, measurement of the stress response at the behavioral level is important. The only known published study utilizing a model of a chronic psychological disorder where animals’ response to stress was validated prior to myocardial ischemic injury is utilizing a predator-based psychosocial model of PTSD (135).

##### A Predator-Based Psychosocial Model of PTSD and Myocardial Ischemia–Reperfusion Injury

A predator-based psychosocial model of PTSD has been utilized to study sensitivity to myocardial ischemic injury. This model involves two 1-h cat exposures, during which rats are restrained while they can see, smell, and hear a cat but cannot be physically harmed. The two exposures are separated by a period of 10 days. Starting on the day of the first cat exposure, rodents experience chronic social instability by having their housing partner changed daily for 31 days. After the 31-day paradigm, rats exhibit a fear memory associated with the cat exposures (evidenced by freezing in response to conditioned context and cues), heightened anxiety-like behavior on the EPM, an exaggerated startle response, and impaired memory for newly learned information. Furthermore, rats exposed to this paradigm have demonstrated physiological changes reflecting elevated SNS activity and HPA axis abnormalities, including elevated heart rate and blood pressure, decreased baseline corticosterone levels, and enhanced negative feedback of the HPA axis (135, 136, 179–181). Replicating and expanding on these results, researchers utilizing this model have shown stressed rats exhibit decreased serotonin, increased norepinephrine, and increased measures of oxidative stress and inflammation in the brain, adrenal glands, and systemic circulation (182, 183).

**Figure 1:**
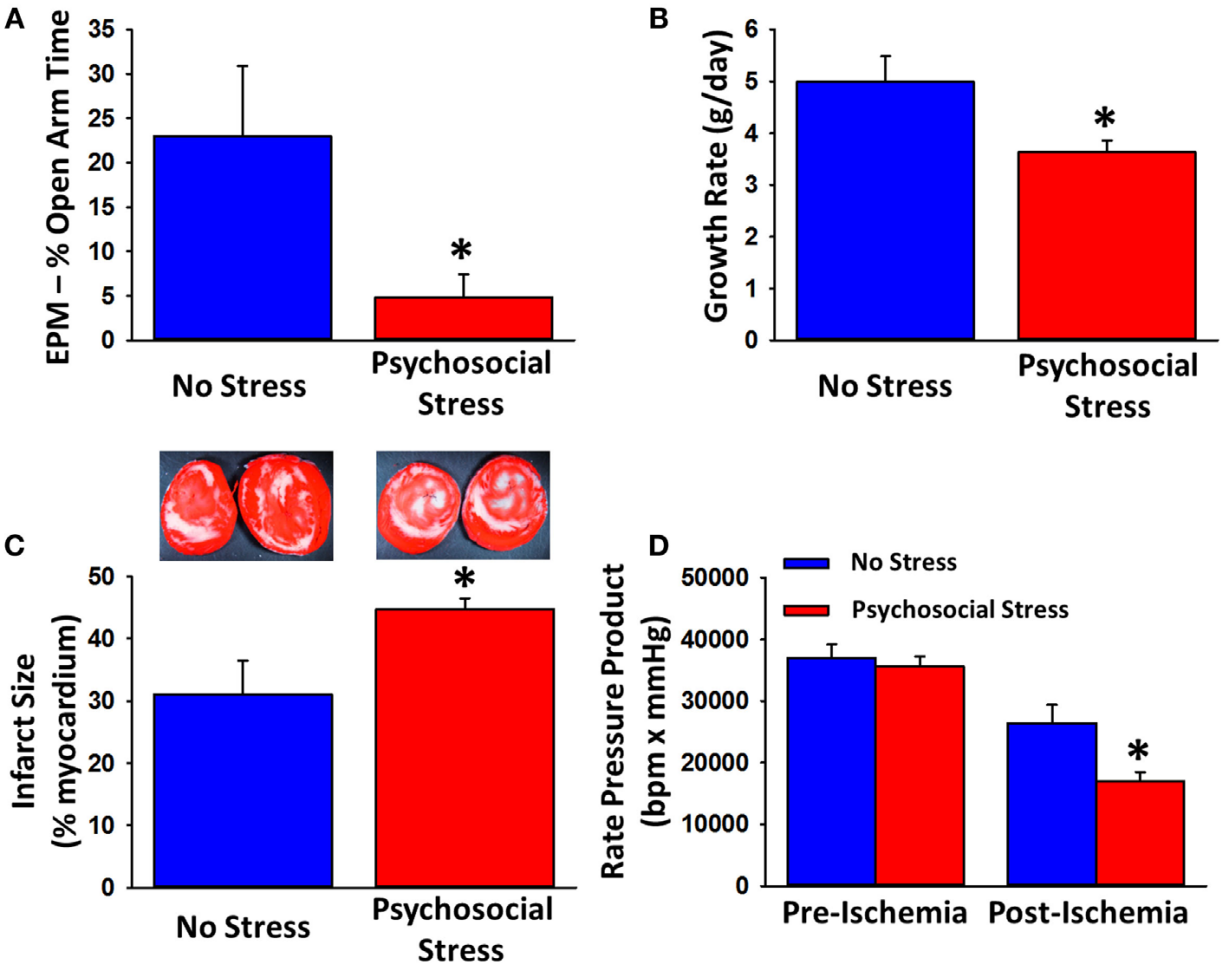
**Effects of a predator-based psychosocial model of PTSD on anxiety-like behavior, growth rate, and myocardial sensitivity to ischemic injury**. Rats exposed to the 31-day psychosocial stress paradigm spend less time in the open arms on the EPM **(A)** and exhibit reduced growth rats **(B)**. Following 20-min ischemia, hearts from psychosocially stressed animals exhibit larger infarcts **(C)**, white regions of representative tissue (samples in the insets) and impaired recovery of contractile function **(D)**. Data are means ± SEM. *p < 0.05 relative to no stress. Adapted from Rorabaugh et al. (135).

Recently, we found that, subsequent to this chronic psychological stress paradigm, male rats exposed to myocardial ischemic injury exhibited greater ISs and decreased recovery of contractile function [Figure 1 (135)]. The disruptive effect of this PTSD paradigm on the heart is further strengthened by anxiety-like behavior in rats on the EPM prior to myocardial ischemic injury. These data suggest that the psychological stress induced by the PTSD paradigm is having an effect directly on the heart, causing the heart to be more susceptible to damage following a MI (135). The ability of chronic stress to worsen the extent of ischemic injury and decrease the recovery of cardiac contractility further exacerbates the supported negative effects of stress in CVD, which make rodents exposed to chronic stress more susceptible to ventricular fibrillation and MI (13, 22, 48, 49, 135).

## The Importance of the Effect of Psychological Stress on Myocardial Ischemia–Reperfusion Injury

Shown presently, acute and chronic psychological stress affects sensitivity to myocardial ischemic injury in opposite directions; acute psychological stress decreases, whereas chronic psychological stress increases sensitivity to myocardial ischemic injury (45, 117). It is possible that protective mechanisms exist in response to an optimal level of acute stress, but these mechanisms are eventually overcome by more intense levels of stress (4).

Physiologically, a possible explanation for this differential effect is that acute psychological stress causes norepinephrine release and acute alpha stimulation, which results in ischemic preconditioning (184, 185). Chronic psychological stress may result in chronic beta stimulation, worsening the ischemic injury (186–190). The previously discussed advantages of the isolated rat heart (66), the wide variety of validated psychological stressors in rodents (119, 122, 123, 136, 174–178), and the existence of rodent models of psychiatric disorders (49, 153, 181) add weight to the presently discussed findings. However, it is important to qualify these findings by recognizing the methodological differences in a limited amount of studies and the previously discussed weaknesses of translating the isolated rodent heart to humans.

Utilizing ethologically valid models of stress to further study the effect of psychological stress on myocardial ischemic injury will best translate to improving patient outcomes in the clinical setting (22, 49). Additionally, further research investigating the effects of stress on the cardiovascular system in females will be important in translating findings to the clinical setting, as the current literature is currently dominated by studies in male subjects (135).

## Conclusion

The relationship between stress and CVD continues to receive a substantial amount of attention. Here, we reviewed research studying the sensitivity of the rodent heart to ischemic injury in response acute and chronic psychological stress in the context of clinical and experimental studies on the effects of stress on the cardiovascular system. Elucidation of stress-limiting systems will help identify novel therapeutic options to decrease cardiovascular mortality. Further research investigating the relationship between acute and chronic stress and ischemic injury will improve patient care with implications that extend beyond cardiovascular disease.

## Author Contributions

EE wrote the first draft of the manuscript and revised it following peer review. BR provided comments on each draft. PZ helped EE prepare the manuscript, provided comments on each draft, and prepared the figures.

## Conflict of Interest Statement

The authors declare that the research was conducted in the absence of any commercial or financial relationships that could be construed as a potential conflict of interest.
